# A Reappraisal of Women's Health Initiative Estrogen-Alone Trial: Long-Term Outcomes in Women 50–59 Years of Age

**DOI:** 10.1155/2015/713295

**Published:** 2015-01-01

**Authors:** Eric Roehm

**Affiliations:** Volunteer Health Clinic, 4215 Medical Pkwy, Austin, TX 78756, USA

## Abstract

The Women's Health Initiative (WHI) Estrogen-Alone Trial randomized postmenopausal women, 50 to 79 years of age, with prior hysterectomy, to conjugated equine estrogens (CEE) or placebo with a 5.9-year median duration of CEE use. In 2013, the WHI published outcomes for additional extended follow-up. Reported here for the first time is an analysis of the number needed to treat with CEE rather than placebo for younger women (50–59 years) to prevent an adverse long-term outcome. For every 76 women randomized to CEE at 50–59 years, one less myocardial infarction occurred during the 13-year cumulative long-term follow-up. For every 37 women randomized to CEE at 50–59 years, one less woman experienced a global index endpoint (including coronary heart disease, invasive breast cancer, stroke, pulmonary embolism, colorectal cancer, hip fracture, and death) during the 13-year follow-up. Younger women (50–59 years), compared to older women, had more favorable cumulative long-term outcomes for MI and global index. Though a subgroup analysis is not an adequate basis for making primary prevention guideline recommendations, the WHI Estrogen-Alone Trial outcomes strongly suggest that a similar course of estrogen initiated at 50–59 years in postmenopausal women with prior hysterectomy results in significant long-term health benefit.

## 1. Introduction 

The Women's Health Initiative (WHI) hormone trials are randomized, double blind, predominantly primary prevention trials, with long-term follow-up evaluating hormone therapy in postmenopausal women. The WHI Estrogen-Alone Trial randomized 10,739 postmenopausal women with prior hysterectomy to either 0.625 mg of conjugated equine estrogens (CEE) daily or placebo [1]. The WHI estrogen plus progestin trial randomized 16,608 postmenopausal women with a uterus to a combination of daily 0.625 mg of CEE and 2.5 mg of medroxyprogesterone acetate or placebo [2]. The strengths of these trials derive, in part, from their large size and long-term follow-up, with a 6.6-year median duration of follow-up in the WHI Estrogen-Alone Trial [3] after the completion of the intervention phase.

However, outcomes resulting from postmenopausal hormone therapy trials may be affected by multiple factors, including the specific estrogen or progestogen agent used, duration of therapy, and characteristics of the group of women treated [4–8]. The inclusion of older women in the WHI trial was recognized as potentially important before any outcomes were reported. The WHI researchers, in a trial design article when discussing the inclusion of older participants in the trial state, “…if the study interventions turn out to be equally efficacious in terms of relative risk reduction throughout the postmenopausal age range… [9]” indicating an awareness by the investigators prior to trial results being available that trial outcomes may vary with age.

The current report makes the case in which outcomes clearly differ by age at time of randomization to CEE in the WHI Estrogen-Alone Trial and that cumulative long-term outcomes for women 50–59 years of age show a net benefit with CEE. In contrast, the WHI trial authors did not take the position in which the evidence from the WHI Estrogen-Alone Trial showed an overall cumulative long-term benefit for younger women randomized to estrogen [3]. Outcome data is presented in detail in the current report to make the case for reinterpretation of the data and to provide a context for outcomes of younger women within the overall trial results for all participants.

## 2. Methods

For the analysis, in this paper, the number needed to treat (NNT) was assessed over the entire 13-year follow-up, including both the intervention and the postintervention phase, in order to help the clinician assess the overall impact of the effect of randomization to CEE in the WHI Estrogen-Alone Trial. The calculations were performed using SAS software (version 9.3) with an add-on module as per Bender [10, 11]. Calculations of the confidence intervals for NNT were calculated based on the Wilson score method as this appears to be the most reliable methodology [10]. The 22% of surviving participants in the WHI Estrogen-Alone Trial who did not consent to extended follow-up [12] are treated as lost to follow-up in the number needed to treat analysis. The number needed to treat provides additional information where the outcomes are initially reported as time to an event [13]. Other *P* values and confidence intervals for hazard ratios in this paper are as provided by the WHI investigators in prior reports.

## 3. WHI Estrogen-Alone Trial

At 40 clinical centers in the United States, the WHI Estrogen-Alone Trial enrolled 10,739 women 50–79 years of age with prior hysterectomy. Subgroup analysis by age was prespecified in the trial protocol [9]. For women at trial entry, 30.9% of participants were 50–59 years, 45.2% of participants were 60–69 years, and 24.0% of participants were 70–79 years of age [3].

The intervention phase lasted for a median duration of 7.2 years before the trial was stopped because of an elevated stroke rate in the CEE group (hazard ratio [HR]: 1.35; 95% nominal confidence interval [CI]: 1.07–1.70) [1, 3]. Median duration of CEE use was 5.9 years [12]. The median adherent time receiving CEE (ingestion of >80% pills) was 3.5 years [12]. The intervention phase plus the postintervention follow-up phase resulted in a cumulative long-term median follow-up of 13 years [3]. A total of 77.9% of surviving participants in the CEE group and 78.4% in the placebo group gave consent for the entire extended follow-up period [12]. The majority of women enrolled in the WHI Estrogen-Alone Trial were without preexisting cardiovascular disease, though, at trial entry, 4.1% had a history of prior myocardial infarction (MI) or coronary revascularization [1].

### 3.1. Age of Starting Estrogen/Placebo

The majority of WHI Estrogen-Alone Trial participants were randomized to CEE or placebo at a median age older than the typical age for starting postmenopausal therapy in clinical practice. The age for initiating CEE or placebo in the trial is shown in Table 1. The estimated median age for randomization to CEE or placebo is 55 years for the 50–59-year group, 64 years for the 60–69-year group, and 74 years of age for the 70–79-year group [1, 3, 14]. A managed care organization reporting on hormone replacement therapy for 1990–1995 showed a median age of 52 years for first time users of hormone replacement therapy [15]. In both the WHI Estrogen-Alone Trial and the WHI estrogen plus progestin trial, the majority of women with a prior history of hormone replacement therapy began at ≤55 years of age. (This can be determined by the previously reported mean age of menopause of trial participants [16, 17] in conjunction with published data on the number of years from menopause at time of starting initial prior course of hormone therapy [18].) Furthermore, a National Health and Nutrition Examination Survey in the United States from 1988 to 1994 reported that the majority of women using hormone replacement therapy started therapy within 1 year of menopause [19].

### 3.2. Outcomes for Women 50–79 Years of Age at Trial Entry

The only statistically significant outcome for all participants (50–79 years) in the WHI Estrogen-Alone Trial for the 13-year cumulative long-term follow-up was a reduction in invasive breast cancer with CEE (HR: 0.79; 95% CI: 0.65–0.97) [3]. There were no other statistically significant differences, including deep vein thrombosis (DVT), stroke, or hip fractures for cumulative long-term follow-up.

In the WHI Estrogen-Alone Trial, for 7.2-year median intervention phase, for all participants (50–79 years), there was no difference with CEE compared to placebo for CHD (coronary heart disease) defined as nonfatal MI or coronary death, the primary trial endpoint (HR: 0.94; 95% CI: 0.78–1.14) [3]. There was an increase in the CEE group CEE in the rate of stroke (HR: 1.35; 95% CI: 1.07–1.70), as well as DVT (HR: 1.48; 95% CI: 1.06–2.07) [3]. Gall bladder disease occurred more frequently with CEE (HR: 1.55; 95% CI: 1.34–1.79) [3, 20]. Hip fractures (HR: 0.67; 95% CI: 0.46–0.96), all fractures (HR: 0.72; 95% CI: 0.64–0.80), and diabetes (HR: 0.86; 95% CI: 0.76–0.98) were less frequent in the CEE group [3]. Of note, the confidence intervals cited throughout this report are for nominal, unadjusted values.

### 3.3. Significant Trends by Age

**Figure 1:**
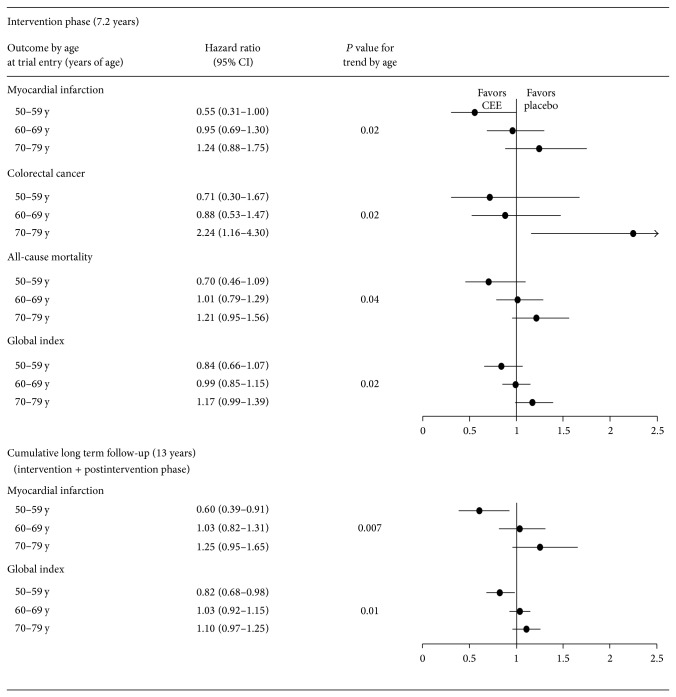
Primary and secondary trial outcomes with a significant trend by age (*P* for interaction) in Women's Health Initiative Estrogen-Alone Trial. CEE: conjugated equine estrogens; y: years of age. Source for outcomes: [3].

Multiple trial outcomes in the WHI Estrogen-Alone Trial were more favorable in younger compared to older participants randomized to CEE. Hazard ratios by decade of age of participant for primary and secondary trial outcomes with a significant trend by age are shown in Figure 1. All outcomes with a statistically significant trend by age had a comparatively more favorable outcome for CEE initiation in younger women compared to older women: myocardial infarction (*P* = .02), total mortality (*P* = .04), colorectal cancer (*P* = .02), and global index (*P* = .02) for the intervention phase and myocardial infarction (*P* = .007) and global index (*P* = .01) for cumulative long-term follow-up [3].

### 3.4. Intervention Phase Outcomes in Women 50–59 Years of Age

For the intervention phase (7.2 years), for women 50–59 years of age in the WHI Estrogen-Alone Trial, there was no statistically significant difference in CEE versus placebo for CHD (HR: 0.60; 95% CI: 0.35–1.04) [3]. There was no statistically significant reduction in MI (HR: 0.55; 95% CI: 0.31–1.00), invasive breast cancer (HR: 0.82; 95% CI: 0.50–1.34), and global index (HR: 0.84; 95% CI: 0.66–1.07) [3]. There was a reduced hazard ratio for CEE compared to placebo for coronary revascularization (HR: 0.56; 95% CI: 0.35–0.88), but the *P* for interaction by age was not statistically significant [3].

For adverse outcomes in the intervention phase, for CEE compared to placebo, in women 50–59 years of age, there was an increase in DVT (HR: 1.66; 95% CI: 0.75–3.67) which is consistent with the increase in DVT for all participants (50–79 years) [3]. Gall bladder disease increased for women 50–59 years of age with CEE (HR: 1.40; 95% CI: 1.10–1.78) [20, 21].

For women 50–59 years of age at trial entry, stroke rate for the intervention phase was similar for CEE and placebo groups (HR: 0.99; 95% CI: 0.53–1.85) [3]. (However, the* P* value (*P* = .77) for trend by age does not suggest that the hazard ratio for the younger age group (50–59 years) can reliably be considered different from the statistically significant elevated hazard ratio for stroke present in the entire group of women 50–79 years of age.)

A post hoc classification of stroke outcomes as ischemic or hemorrhagic showed a HR of 1.09 (95% CI, 0.54–2.21) for women 50–59 years of age randomized to CEE for ischemic strokes [22]. For women less than 10 years post menopause, the risk of ischemic stroke for CEE was increased (HR: 2.62; 95% CI: 1.01–6.81) during the intervention phase [22]. Another WHI report indicated that the increase in total stroke seen with women less than 10 years from menopause was attenuated when women with prior cardiovascular disease and >60 years were excluded (HR 1.23 versus 1.77) from an analysis of the combined WHI trials [23].

### 3.5. Long-Term Outcomes with a Hazard Ratio of Less than 1.0 in Women 50–59 Years of Age

With cumulative long-term follow-up of 13 years in the WHI Estrogen-Alone Trial, there were a number of outcomes with a reduced hazard ratio for CEE versus placebo in women 50–59 years of age at trial entry [3], Table 2. The hazard ratios comparing CEE to placebo were reduced for CHD (HR: 0.65; 95% CI: 0.44–0.96), myocardial infarction (MI) (HR: 0.60; 95% CI: 0.39–0.91), all cancer types (HR: 0.80; 95% CI: 0.64–0.99), and global index (HR: 0.82; 95% CI: 0.68–0.98) [3]. However, the outcomes of myocardial infarction and global index were the only outcomes with both a reduced hazard ratio and a statistically significant p for interaction for age for CEE versus placebo [3]. Invasive breast cancer was decreased with CEE for long-term follow-up in women 50–59 years of age (HR: 0.76; 95% CI: 0.52–1.11) similar to the statistically significant reduction in invasive breast cancer occurring for all participants 50–79 years of age randomized to CEE (HR: 0.79; 95% CI: 0.65–0.97) [3].

### 3.6. Magnitude of Favorable Long-Term Outcomes in Women 50–59 Years of Age

**Figure 2:**
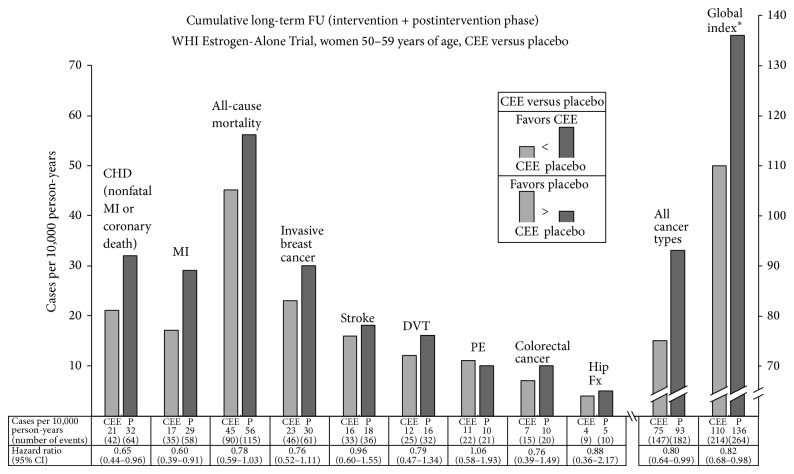
Outcomes for women 50-59 years of age at trial entry, conjugated equine estrogens (CEE) versus placebo, cumulative long-term follow-up (13 years), and WHI Estrogen-Alone Trial. ∗ Global index represents the first event for each participant from among the following: coronary heart disease (nonfatal MI or coronary death), stroke, pulmonary embolism, breast cancer, colorectal cancer, hip fracture, or death due to other causes. CEE: conjugated equine estrogens; CHD: coronary heart disease; CI: confidence interval; DVT: deep vein thrombosis; FU: follow-up; Fx: fracture; MI: myocardial infarction; P: placebo; PE: pulmonary embolus; WHI: Women's Health Initiative. Source for outcomes: [3].

Annualized incidence rates allow direct comparison of the magnitude of the difference between CEE and placebo groups, while hazard ratios provide information on the relative difference in outcome, but not the absolute differences in outcome. In Figure 2, annualized incidence rates as well as hazard ratios [3] for the cumulative 13-year long-term follow-up in WHI Estrogen-Alone Trial are shown in graph form for the first time for the 50–59-year group with an adequate scale to allow a comparative assessment of the magnitude of the difference between CEE versus placebo for multiple outcomes.

### 3.7. Number Needed to Treat to Avoid Adverse Outcome in Women 50–59 Years of Age

The number of women needed to be randomized to CEE to prevent one woman from developing an adverse outcome during the cumulative 13-year follow-up in the WHI Estrogen-Alone Trial, for women 50–59 years of age, was calculated as noted in the Methods Section. This number-needed-to-treat analysis provides a measure of the summation effect of randomization to CEE versus placebo for the entire 13-year cumulative follow-up period. For every 76 (95% CI, 40.3–497.2) women randomized to CEE rather than placebo at 50–59 years of age, there was one less woman having a myocardial infarction (MI) (Table 3). For every 37 (95% CI, 19.6–312.6) women randomized to CEE rather than placebo at 50–59 years of age, there was one less woman who developed a global index endpoint (coronary heart disease, invasive breast cancer, stroke, pulmonary embolus, colorectal cancer, hip fracture, and death from other causes).

A number-needed-to-treat analysis for MI and global index for the intervention phase for women 50–59 years of age did not show a statistically significant difference in outcomes for the CEE versus placebo group.

### 3.8. WHI Estrogen-Alone Trial and Breast Cancer Reduction

In the WHI Estrogen-Alone Trial with cumulative long-term follow-up, CEE use for a 5.9-year median duration compared to placebo resulted in a statistically significant reduction in invasive breast cancer for women 50–79 years of age (HR: 0.79; 95% CI: 0.65–0.97) [3]. Breast cancer risk reduction was concentrated in women without benign breast disease or family history of breast cancer [24]. A sensitivity analysis showed that better adherence to estrogen use within the trial was associated with a lower risk of invasive breast cancer [24]. Women randomized to CEE compared to placebo were less likely to die of breast cancer (HR: 0.37; 95% CI: 0.13–0.91) and fewer women died from any cause after a breast cancer diagnosis (HR: 0.62; 95% CI: 0.39–0.97) [24]. In contrast, the WHI estrogen plus progestin trial for women 50–79 years of age showed an increase in invasive breast cancer with long-term follow-up (HR: 1.28; 95% CI: 1.11–1.48) [3], with the addition of 2.5 mg of medroxyprogesterone acetate daily to the same dose of CEE. The increased breast cancer risk was initially concentrated in women with prior postmenopausal hormone use [25]. The similar incidence rates of breast cancer for the placebo groups in the two trials suggest that the difference in outcome between the trials is primarily the result of the addition of medroxyprogesterone acetate to CEE [24].

### 3.9. WHI Trial Results Stratified by Years Since Menopause

**Figure 3:**
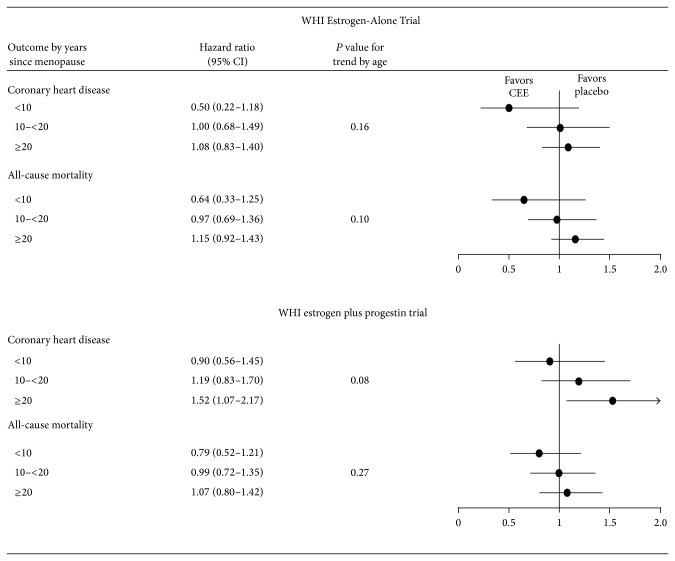
Intervention phase outcomes by years since menopause at trial entry of WHI Estrogen-Alone Trial and WHI estrogen plus progestin trial. CEE: conjugated equine estrogens; WHI: Women's Health Initiative. Sources for outcomes: [3, 23].

Outcome data stratified by years since menopause [3, 23] has been published for the WHI Estrogen-Alone Trial and WHI estrogen plus progestin trial for the intervention phase (Figure 3). In both trials, there was a trend for better CHD and mortality outcomes with hormone therapy when comparing women closer to menopause with women farther from menopause.

There is a high degree of biologic plausibility that a woman's age and duration of time since menopause at initiation of hormone replacement therapy may affect clinical outcome [26]. The timing hypothesis, which is supported by primate work [27], proposes that hormone replacement therapy may have an adverse effect when begun late in menopause contrasting with beneficial effects on the more normal vessels typically present in younger women closer to time of menopause [26]. A WHI substudy of women 50–59 years of age showed significantly less coronary artery calcification, a quantitative marker for atherosclerotic plaque, in the CEE group compared to placebo [16]. The Kronos early estrogen prevention study (KEEPS) involved a younger, healthier group of newly menopausal women who developed minimal disease over the treatment period of 4 years [28]. KEEPS showed no statistically significant difference in coronary calcification or carotid artery intima-media thickness scores for women treated with estrogen and progesterone compared to the placebo group [28].

## 4. Long-Term Follow-Up for Clinical Outcomes Is Optimal

The WHI Estrogen-Alone Trial results indicate that long-term follow-up is required to fully assess the effects of estrogen in postmenopausal women. For all participants (50–79 years) in the WHI Estrogen-Alone Trial, a reduction in invasive breast cancer only became statistically significant with approximately an additional 5 years of follow-up after the completion of the CEE intervention phase [1, 12]. Similarly, in women 50 to 59 years of age, there were nonsignificant trends for a reduction in MI and global index in the CEE group at the completion of the 7.2-year intervention phase that only became statistically significant after years of additional follow-up subsequent to the completion of the CEE intervention phase [3, 12].

The single published Kaplan-Meier estimate for women 50–59 years of age (known to the author) from the WHI Estrogen-Alone Trial is for the outcome of CHD for the intervention phase of the trial [29]. The Kaplan-Meier curves of cumulative hazard for CHD in regard to CEE versus placebo for the intervention phase showed a divergence developing with time over the median of 7.2 years of follow-up [29]. In Table 4, outcome events and relative risk for both the intervention and postintervention phase of the trial are shown for CHD, MI, death, and global index using data derived from prior WHI publications [3, 12]. Given the data as shown in Table 4, diverging curves for CHD for the intervention phase would persist and maintain a pattern of diverging curves when followed beyond the end of the intervention phase. The data for MI, death, and global index for women 50–59 years of age would also show diverging curves extending beyond the intervention phase if presented as a Kaplan-Meier estimate of cumulative hazard.

**Figure 4:**
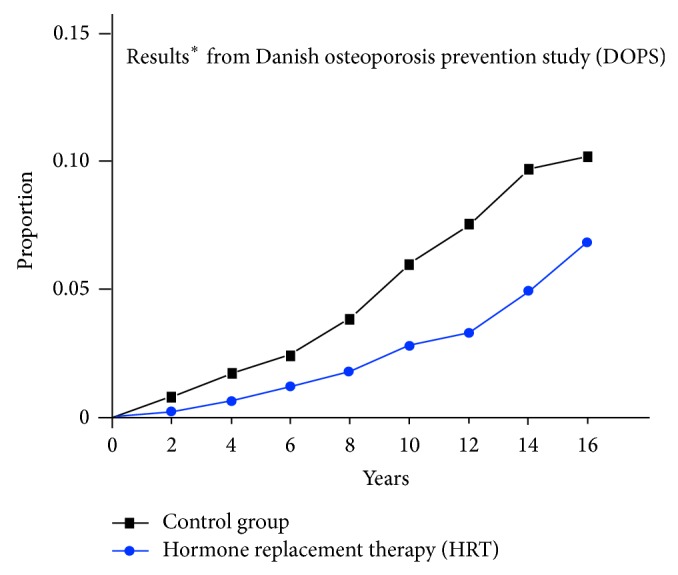
Cumulative hazard of developing death or hospitalization due to heart failure or myocardial infarction. ^*^Modified from Schierbeck [30].

The only published Kaplan-Meier estimate extending at least 10 years for hormone replacement therapy for women less than 60 years of age in a primary prevention trial is from the Danish osteoporosis prevention study (DOPS) [30]. Diverging outcomes extending past the 10-year drug intervention were shown in DOPS, comparing hormone therapy (estradiol plus or minus norethisterone acetate, started within 2 years of menopause) to no medication, for the combined endpoint of death or hospital admission for heart failure or MI in this randomized, unblinded study [30] (Figure 4).

If Kaplan-Meier cumulative hazard estimates extending through 10 years had been published for the WHI Estrogen-Alone Trial for CHD, death, MI, or global index endpoints for women 50–59 years of age, similar diverging outcome curves to the DOPS data would be shown. Long-term follow-up of cardiovascular, cancer, and mortality outcomes through 10 years, including at least 5 years of follow-up after completion of hormone therapy, is advisable to adequately assess the effects of hormone replacement therapy.

Of note, in DOPS, where 81% of the women received both estrogen and progestin, participants had predominantly favorable outcomes [30], while women 50–59 years of age in the WHI estrogen plus progestin trial had negative outcomes or trends for coronary artery disease, stroke, deep venous thrombosis, and pulmonary embolism [3]. The difference between the trials may be due in part to the very different populations in the trials (younger women, ≤2 years from menopause and with lower cardiovascular risk profiles in DOPS), as well as the particular hormone therapy and duration of therapy used in the trials, though this remains conjecture.

### 4.1. Factors Affecting Long-Term Outcomes

The long duration of the follow-up of the WHI Estrogen-Alone Trial may have influenced outcomes. The 13-year median cumulative follow-up included the intervention phase and the 6.6-year median duration postintervention phase of the trial. The WHI trial study's authors reported on that time duration for their most recent comprehensive article on outcomes [3] and, hence, that time duration was used in this paper.

The initial adverse effect of CEE on stroke and DVT documented during the intervention phase that diminished in the postintervention phase may have been simply the waning of the adverse effect that occurred after CEE was discontinued. The similarity of the relative risks in the intervention and postintervention phase for myocardial infarction, CHD, death, invasive breast cancer, and global index suggests a possible perseverance of a beneficial effect of CEE that lasted beyond cessation of the medication. If vascular beneficial effects occurred during the intervention phase, the ramification of these effects would tend to continue to manifest beyond the point in time when CEE was discontinued. The mechanism of the persistent effects of hormone therapy after cessation in regard to invasive breast cancer is beyond the scope of this paper.

The unblinding occurred when the trial was stopped because of an increased risk of stroke in the CEE group [1]. This would not tend to lead to preferential reporting of adverse events in the placebo group participants during the postintervention follow-up. Hence, the unblinding of the trial in the post intervention phase is unlikely to bias the reporting of adverse outcomes in favor of the CEE group.

## 5. Age as an Important Subgroup

Age was prespecified in the WHI trial protocol as a subgroup for analysis. A limited number of subgroup analyses in the WHI hormone trial reports were thought to be important enough to warrant stratifying primary outcomes in the presentation of the initial WHI trial results [1, 2]. In the WHI estrogen plus progestin trial 2002 publication, there were four such subgroups: clinical center, age, prior to disease, and randomization status in the low-fat diet trial [2]. Similarly, in the WHI Estrogen-Alone Trial initial 2004 publication, primary outcome comparisons were presented as hazard ratios from Cox proportional hazard analyses stratified by only three subgroups: age, prior disease, and randomization status in the low-fat diet trial [1]. Age was an important consideration in the trial, with the protocol defining specific target age enrollment percentages [9]. Though a subgroup analysis of multiple biomarkers obtained from blood draw analysis in a nested case control study was also prespecified by protocol, this analysis was reported only after initial publication of trial results [29, 31, 32]. The biomarker subgroup analysis was performed for a comparatively limited number of outcomes [3, 12, 29, 31, 32].

## 6. Limitations of This Analysis

This paper concerns a subgroup analysis of women 50–59 years of age at trial entry in the WHI Estrogen-Alone Trial. Subgroup analyses are subject to statistical problems: limiting conclusions for subgroup analyses to those with a statistically significant p for interaction is helpful. A limitation of this analysis is that CHD, the primary trial outcome, did not have a statistically significant *P* for interaction (*P* = .08 for intervention phase [3]). This trial also reflects the outcomes for only a single formulation of estrogen: CEE.

Multiple primary and secondary endpoints underwent evaluation for trend by age (*P* for interaction). Four primary or secondary endpoints in the intervention phase and two in the cumulative long-term follow-up had a significant trend by age at the .05 significance level. For the 20 primary and secondary trial endpoints [3] in the WHI Estrogen-Alone Trial undergoing analysis by age, one significant *P* for interaction would be expected by chance alone in the intervention phase and one by chance alone for cumulative long-term follow-up at the .05 level of significance.

The number-needed-to-treat analysis includes the extended follow-up phase which is unblinded and with incomplete participant follow-up. These same limitations are present for all WHI reported annualized incidence percentages and rates for postintervention and cumulative long-term follow-up [3, 12, 24, 33, 34].

Nominal 95% confidence intervals are reported without adjustment for multiple outcomes, sequential repeated data analysis, multiple subgroup analyses, and extension of trial follow-up with incomplete participation. This is true for this report and for the majority of the published WHI trial outcome results [3, 12, 20, 24, 32–34]. There has been no publication from the WHI Estrogen-Alone Trial of a primary or secondary trial endpoint fully adjusted for these factors (except for a benefit with CEE for all fractures [1]) which achieves statistical significance for any follow-up interval, including stroke or DVT in women 50–79 years of age [1, 3, 12, 23, 24, 29, 32].

## 7. Conclusions

Women 50–59 years of age at time of randomization to CEE or placebo in the WHI Estrogen-Alone Trial were similar in median age to women initiating hormone replacement therapy in clinical practice. In the intervention phase, for women 50–59 years of age with CEE, there was an increased risk of DVT, gall bladder disease, and stroke, while the reduction in MI, invasive breast cancer, and global index of events was not statistically significant. With cumulative 13-year long-term follow-up, women 50–59 years of age with CEE showed a reduction in MI, as well as a reduced global index of events. The increased risk of stroke and DVT in the intervention phase for women 50–79 years of age, which did not show significant trends with age, declined with cessation of CEE. Long-term follow-up including at least 5 years of follow-up after completion of hormone therapy is necessary to optimally evaluate effects of hormone replacement therapy on cardiovascular, cancer, and mortality outcomes. Though a subgroup analysis does not provide an adequate basis for making guideline recommendations for primary prevention, the preponderance of evidence in the WHI Estrogen-Alone Trial strongly suggests an overall benefit with CEE with cumulative long-term follow-up in women 50–59 years of age. These potential benefits only apply to women with prior hysterectomy and for duration of CEE use similar to what was used in the trial. The WHI Estrogen-Alone Trial data does not provide information on longer durations of use and strongly suggests that initiation of hormone therapy at significantly later ages is harmful.

## Figures and Tables

**Table 1:** Women's Health Initiative Estrogen-Alone Trial, age of participants in estrogen/placebo (intervention) phase of trial.

Age at trial entry [3] Years (%, Number)	Age at end of CEE/placebo intervention phase^a^	Median age at randomization to CEE/placebo^b^	Median age of starting postmenopausal hormone replacement therapy in USA population [15–19]: <55 years of age
50–59 y (30.9%^c^, 3313)	55–67	55	
60–69 y (45.2%, 4851)	65–77	64	
70–79 y (24.0%, 2575)	75–87	74	

^a^A minority of participants were older at the end of the intervention phase than those listed in the upper limits of the estimated age brackets. (Start of intervention phase: December 1993; end of enrollment for intervention phase: October 1998; end of intervention phase: February 29, 2004; median duration of intervention phase: 7.2 years; end of reported trial follow-up: September 30, 2010 [1, 3].)

^b^Estimates adjusted for age distribution of 50–59 y group (50–54 y/55–59 y = 1 : 1.37 ratio [14]) and for possible enrollment of participants at lower end of age bracket.

^c^Percentages do not add to 100% because of rounding anomaly.

CEE: conjugated equine estrogens; USA: United States of America.

Derived from data provided in [1, 3, 14–19].

**Table 2:** Women 50–59 years of age at trial entry: WHI Estrogen-Alone Trial outcomes, 13-year cumulative long-term follow-up (intervention phase + postintervention phase).

Outcome	Hazard ratio (95% CI) Women 50–59 years of age CEE versus placebo	*P* for interaction (trend by age) for all participants, 50–79 years
Coronary heart disease (primary trial endpoint)^a^	0.65 (95% CI, 0.44–0.96)^†^	0.12
Myocardial infarction	0.60 (95% CI, 0.39–0.91)^†^	0.007^‡^
Invasive breast cancer	0.76 (95% CI, 0.52–1.11)	0.70
All cancer types	0.80 (95% CI, 0.64–0.99)^†^	0.18
All-cause death	0.78 (95% CI, 0.59–1.03)	0.10
Global index^b^	0.82 (95% CI, 0.68–0.98)^†^	0.01^‡^

^a^Coronary heart disease: nonfatal myocardial infarction or coronary death.

^b^Global index represents the first event for each participant from among the following: coronary heart disease, stroke, pulmonary embolism, invasive breast cancer, colorectal cancer, hip fracture, or death due to other causes.

^†^95% confidence interval does not include 1.0.

^‡^Statistically significant (95% CI) *P* for interaction (trend by age).

CEE: conjugated equine estrogens; CI: confidence interval; WHI: Women's Health Initiative.

Source for outcomes: [3].

**Table 3:** Number of women needed to treat (randomized to CEE) to prevent one woman from developing a myocardial infarction or global index event, women aged 50–59 years at trial entry, Women's Health Initiative Estrogen-Alone Trial, and cumulative long-term outcomes.

Cumulative long-term (13-year median duration) outcomes		
(intervention phase + postintervention phase)		
Outcome	Number needed (95% CI) to treat	Effect
Myocardial infarction	76 (40.3–497.2)	1 less woman with a myocardial infarction
Global index^a^	37 (19.6–312.6)	1 less woman with a global index event

A number needed to treat analysis for MI and global index for the intervention phase for women aged 50–59 years did not show a statistically significant difference in outcomes for the CEE versus placebo group.

^a^A participant is counted as having a global index event if there is the diagnosis of one or more of the following occurring after randomization: coronary heart disease (nonfatal MI or coronary death), stroke, pulmonary embolism, invasive breast cancer, colorectal cancer, hip fracture, or death due to other causes.

CEE: conjugated equine estrogens; CI: confidence interval; MI: myocardial infarction; NS: not significant.

Derived (as per Methods Section) from data provided in [3].

**Table 4:** Women 50–59 years of age at trial entry, WHI Estrogen-Alone Trial Outcomes, intervention phase and postintervention phase, events, and relative risk.

Outcome	Intervention phase			Postintervention phase		
	Events and relative risk (RR)^†^			Events and relative risk (RR)^†^		
	CEE *N* = 1639	Placebo *N* = 1674	RR^†^ (95% CI)	CEE *N* = 1223	Placebo *N* = 1232	RR^†^ (95% CI)
	Events			Events		
CHD	21	35	0.61 (0.36–1.05)	21	29	0.73 (0.42–1.27)
MI	17	31	0.56 (0.31–1.01)	18	27	0.67 (0.37–1.21)
Death	35	50	0.71 (0.47–1.10)	55	65	0.85 (0.60–1.21)
Global index^a^	117	142	0.84 (0.67–1.06)	97	122	0.80 (0.62–1.03)

^†^Hazard ratios in WHI Hormone Trials apply a time to event analysis which can not be duplicated without full access to the data set. Relative risks in this table are calculated and shown (no time to event analysis) to allow comparison of the intervention phase and post intervention phase data.

^a^Global index represents the first event for each participant from among the following: coronary heart disease, stroke, pulmonary embolism, invasive breast cancer, colorectal cancer, hip fracture, or death due to other causes.

CEE, conjugated equine estrogens; CHD, coronary heart disease; CI, confidence interval; MI, myocardial infarction; RR, relative risk; WHI, Women's Health Initiative.

Source for the number of events and number of participants: [3, 12].
